# Public perceptions and emotional trends of psychotherapy: a 15-year analysis across English and Spanish language communities on X (twitter)

**DOI:** 10.3389/fdgth.2025.1598237

**Published:** 2025-12-16

**Authors:** Javier Goena, Juan Pablo Chart-Pascual, Francisco J. Lara-Abelenda, David Chushig-Muzo, Maria Montero-Torres, Ana Gonzalez-Pinto, Guillermo Lahera, Ana Catalan, Miguel Angel Gonzalez-Torres, Mariana Pinto da Costa, Melchor Alvarez-Mon, Miguel Angel Alvarez-Mon

**Affiliations:** 1 Psychiatry Department, Basurto University Hospital, Osakidetza Basque Health Service, Bilbao, Spain; 2 Biobizkaia Health Research Institute, OSI Bilbao-Basurto, Bilbao, Spain; 3 Psychiatry Department, Araba University Hospital, Osakidetza Basque Health Service, Vitoria-Gasteiz, Spain; 4 University of the Basque Country UPV/EHU, Vitoria-Gasteiz, Spain; 5 Bioaraba Health Research Institute, Vitoria-Gasteiz, Spain; 6 CIBERSAM, Madrid, Spain; 7 Psychiatry Service, Center for Biomedical Research in the Mental Health Network, University Hospital Príncipe de Asturias, Alcalá de Henares, Spain; 8 Department of Signal Theory and Communications and Telematic Systems and Computing, School of Telecommunications Engineering, Rey Juan Carlos University, Madrid, Spain; 9 Department of Medicine and Medical Specialties, University of Alcala, Alcalá de Henares, Spain; 10 Ramón y Cajal Institute of Sanitary Research (IRYCIS), Madrid, Spain; 11 Neuroscience Department, University of the Basque Country, Leioa, Spain; 12 Department of Psychosis Studies, Institute of Psychiatry, Psychology & Neuroscience, King’s College London, London, United Kingdom; 13 Institute of Biomedical Sciences Abel Salazar, University of Porto, Porto, Portugal; 14 Immune System Diseases-Rheumatology, Oncology Service an Internal Medicine, University Hospital Príncipe de Asturias (CIBEREHD), Alcalá de Henares, Spain; 15 Department of Psychiatry and Mental Health, Hospital Universitario Infanta Leonor, Madrid, Spain

**Keywords:** X, twitter, psychotherapy, psychoanalysis, cognitive behavioral therapy, acceptance and commitment therapy, narrative therapy, natural language processing

## Abstract

**Background:**

Mental health disorders have been rising globally, and social media platforms provide a unique opportunity to examine public perceptions of psychotherapy. However, little is known about how different therapeutic modalities are discussed across linguistic and cultural contexts.

**Objective:**

To analyse how psychotherapies are discussed on X (formerly Twitter) over a 15-year period, comparing thematic content and emotional tone between English and Spanish-speaking communities' tweets.

**Methods:**

We collected 102,946 public tweets from 2008 to 2022, including 76,878 in English (74.7%) and 26,068 in Spanish (25.3%), related to four therapies: Acceptance and Commitment Therapy (ACT), Cognitive Behavioural Therapy (CBT), Psychoanalytic/Psychodynamic Therapy (PAT), and Narrative Therapy (NT). Topic modelling was performed using BERTopic. Emotion classification was conducted using DistilRoBERTa and Robertuito pre-trained transformer-based language models based on Ekman's six basic emotions.

**Results:**

CBT was the most frequently mentioned therapy (51,250 tweets, 49.8%), followed by ACT (18,196 tweets, 17.7%). In English tweets, the main theme was professional therapy promotion (CBT: 29,383 tweets), and fear was the most prevalent emotion. In Spanish tweets, personal experiences were more common, particularly in ACT (2,528 tweets), while anger dominated tweets about PAT (2,825 tweets), linked to a lack of understanding. Joy emerged as the dominant emotion in Spanish tweets about ACT and NT. The volume of tweets increased notably from 2020 onwards, especially in English, coinciding with the COVID-19 pandemic.

**Conclusions:**

Clear linguistic differences shape the public discourse around psychotherapy. English tweets emphasise clinical utility and are marked by fear, whereas Spanish tweets focus on individual experiences, with emotions ranging from joy to frustration. These findings underscore the importance of culturally adapted mental health communication strategies on social media.

## 1 Introduction

Over the past few decades, there has been a steady rise in the prevalence of mental health disorders, including anxiety, depression and suicide (1). This increase can be attributed to a variety of factors, such as intense workplace pressures, pervasive use of digital technologies and social isolation, which have compounded the mental strain on individuals globally (2–4). Even before the COVID-19 pandemic, high levels of anxiety and stress were already evident in various countries, with mental health services struggling to meet the growing demand. These long-standing issues, however, were exacerbated by the pandemic, highlighting existing deficiencies in mental health services (5). According to the World Health Organization (WHO), the first year of the pandemic saw a 25% increase in the global prevalence of these conditions (6). While the pandemic significantly intensified mental health challenges, it also underscored the urgent need for enhanced mental health support, which had been mounting prior to the crisis. The disruption to mental health services in 93% of countries during the pandemic further widened the gap between mental health needs and available care (7–9).

Simultaneously, there has been a positive shift towards greater recognition and accessibility of mental health resources (10). The stigma associated with seeking help for mental health issues has diminished, leading to more people coming forward to seek aid (11). The proliferation of digital platforms has played a pivotal role in this change, making mental health information more accessible and fostering supportive communities online (12). Moreover, telehealth, which saw rapid adoption during the pandemic, has emerged as a vital service model, expanding access to mental health care (13–16).

Psychotherapy has long been recognised as a primary tool in treating anxiety disorders and related conditions (17). The efficacy of different types and schools of psychotherapy has been the subject of numerous studies, aiming to identify which approaches are most effective for specific disorders and populations (18–20). Research has explored various psychotherapeutic modalities, such as Cognitive Behavioural Therapy (CBT) and third-wave CBTs (21, 22), Psychodynamic Therapy (23, 24), and Interpersonal Therapy. Studies typically measure outcomes such as reduction in symptom severity, improvement in quality of life and rates of remission (25). However, these studies face several limitations, including small sample sizes, lack of control groups and short follow-up periods, which can affect the reliability of their findings (26). Psychotherapy is highly individualised, making it difficult to standardise treatments across studies or to apply findings universally (27). The subjective nature of psychological symptoms and improvements poses challenges for objectively measuring treatment success (28, 29). In fact, beyond specific techniques or modalities, common factors such as therapeutic alliance, empathy, and expectations towards the therapy are responsible for change (30, 31).

Given the complex nature of psychotherapy and its outcomes, qualitative research becomes essential (32, 33). Qualitative studies can provide deeper insights into the experiences of individuals undergoing therapy, the contextual factors influencing therapy's success, and the practical aspects of implementation and preventive measures (34, 35). These studies are invaluable for understanding how psychotherapy can be adapted and optimised for different populations and settings.

In the current landscape of mental health research, there remains a significant gap in understanding how effective people think psychotherapies are, as discussed on social media platforms, particularly X. While numerous studies have evaluated the clinical effectiveness of various psychotherapeutic approaches, fewer have focused on the public's perceived efficacy—how people believe these therapies work for them based on anecdotal and shared experiences online. This perceived efficacy is crucial as it can influence both the willingness to seek therapy and the expected outcomes of such treatments (80).

Moreover, the stigma associated with seeking mental health treatment continues to be a pervasive barrier. Social media, with its vast reach and influence, plays a dual role in either perpetuating this stigma or potentially dismantling it by normalising mental health discussions. Analysing conversations on platforms like X, Facebook, Instagram or TikTok, could provide valuable insights into prevailing attitudes and may help identify specific misconceptions or supportive elements that could be targeted to reduce stigma (36–38).

The digital age has significantly lowered the barriers to sharing information, resulting in a proliferation of mental health advice from sources that often lack proper credentials. This can lead to widespread misinformation, which may confuse or mislead individuals seeking genuine psychological help (39, 40). Analysing social media discussions can reveal how misinformation influence public opinions and potentially erode trust in established, evidence-based psychotherapy methods (41).

Although some initial studies have evaluated different perspectives and points related to psychotherapy on X (42), the broader social perspectives, especially cross-cultural and multilingual insights, remain understudied. Social and cultural attitudes play a critical role in shaping public perception, perceived efficacy, and overall success of therapy, yet there is a notable gap in research examining these factors across diverse linguistic and cultural contexts. By aligning public perceptions with clinical practices, mental health professionals can better tailor their approaches to meet the needs and expectations of the community (43). This study uniquely tries to address this gap by applying advanced artificial intelligence techniques to analyse public discussions on X in both English and Spanish. By doing so, we aim to shed light on the social and cultural factors that influence the real effectiveness of psychotherapy.

## 2 Material and methods

### 2.1 Study design

We designed an observational study to analyse tweets related to different types of psychotherapies. The therapies selected—Acceptance and Commitment Therapy (ACT), Cognitive Behavioural Therapy (CBT), Psychoanalytic and Psychodynamic Therapy (PAT), and Narrative Therapy (NT)—represent a broad spectrum of psychological approaches. These were chosen for their diverse methodologies, ranging from structured methods like ACT or CBT, to more interpretative styles such as Psychoanalytic and Narrative therapies. These therapies are not only clinically significant but also frequently discussed on social media, indicating substantial public interest and engagement (42). Other evidence-based approaches, such as Mindfulness-Based Therapy or Interpersonal Therapy, were initially considered. However, these terms produced a high proportion of non-clinical or ambiguous tweets (e.g., general lifestyle advice), which limited their suitability for NLP-based classification.

X was selected as the data source due to its large volume of publicly accessible content, allowing for a more comprehensive analysis of psychotherapy discussions. Its text-based format also enables the application of Natural Language Processing (NLP) techniques for nuanced sentiment and thematic analysis.

The research protocol received approval from the Ethics Committee of the University of Alcalá, ensuring compliance with the ethical standards delineated in the Declaration of Helsinki. The study design ensures that no human subjects are involved directly, and confidentiality is maintained as neither personal data related to the tweet authors nor any potentially identifying information is disclosed.

### 2.2 X data acquisition and search strategy

Tweets were collected in both Spanish and English, covering a period from 2008 to 2022. The 15-year period was chosen to capture the long-term evolution of psychotherapy discourse on X, including shifts in perceptions influenced by key events like the platform's growth, increased mental health discussions, and the impact of the COVID-19 pandemic.

English was chosen as the primary language due to its widespread use on X, offering the largest and most representative dataset for global psychotherapy discussions. Spanish was selected to complement this with a linguistically and contextually distinct sample, allowing for the exploration of how psychotherapy is discussed in another major world language, which may reflect different traditions of clinical practice and social discourse.

We utilised the Twitter Binder search engine. This tool was selected for its capabilities to perform comprehensive searches based on specific keywords, allowing us to access 100% of public tweets. Tweet Binder captures all tweets containing the selected keywords, not only when they appear as hashtags. In some of our previous social media studies, we relied on specific hashtags when these represented the dominant form of online discourse around the topic under investigation. In the present study, however, this approach would have excluded a substantial number of relevant tweets, as psychotherapies are frequently referred to in plain text (e.g., cognitive behavioral therapy, terapia cognitivo-conductual) and not consistently accompanied by hashtags. For this reason, we searched directly by the names of the psychotherapies in both English and Spanish, ensuring that all relevant mentions were included regardless of hashtag use. The complete list of keywords used in the data collection process is provided in the Supplementary Material (Supplementary Section S1).

### 2.3 Data analysis

The preprocessing of tweets was executed using Python's NLP libraries. This step involved cleaning the data, normalising the text, and classifying tweets by language, which was fundamental for tailored analytic processes.

Advanced artificial intelligence techniques, including machine learning, deep learning, and NLP, were employed to dissect and understand the themes and nuances within the tweet corpus. The use of artificial intelligence for social media infodemiology has been extensively used in the literature (44–46), employing two types of machine learning analysis: supervised and unsupervised (47). The primary analytical approach was unsupervised learning, which allowed for the identification of inherent patterns and themes without the need for pre-labeled training data. This method is particularly suited to exploratory data analysis where the categorization of themes evolves organically from the data itself.

Using unsupervised analysis, we aimed to detect hot topics and conversations in social media related to the selected psychotherapies. To detect these topics, we followed the methodology developed by Maarten Grootendorst called BERTopic (48). The first step is to convert the text into a numerical vector through a process known as vectorization. Although there are several text vectorisation methods, we used a transformer called Sentence-BERT (49). Sentence-BERT, a modification of BERT (50), specialises in creating vectors that maintain contextual relationships while reducing computation time. The vectors generated by Sentence-BERT have a predefined length of 768 numbers. Given the high dimensionality of these vectors, we need to create a smaller and denser vector that retains the underlying structures. The method used to reduce the length is UMAP (Uniform Manifold Approximation and Projection), a non-linear dimensionality reduction algorithm used in the exploratory analysis of high-dimensional data (51). After reducing the vectors, we identified groups of samples using a clustering algorithm. In this project, we use the Hierarchical Density-Based Spatial Clustering of Applications with Noise (HDBSCAN) model (52), which uses sample densities to identify closely related sets while separating them from others, classifying them as groups. HDBSCAN is commonly used with vectors extracted from UMAP because both consider topological factors (53). An additional advantage of using HDBSCAN is that, unlike other clustering algorithms, it automatically determines the number of groups to detect without requiring a manual selection.

No explicit filtering of topics was applied after the topic modelling; however, we adjusted a key BERTopic hyperparameter—the minimum number of tweets required to form a new cluster—in order to avoid generating a hug eamount of small overlapping topics. This adjustment allowed us to focus on the most salient and coherent topics for analysis.

Once the tweets were classified into various groups, it was necessary to determine the common themes among the samples in each group. To do this, we performed thorough preprocessing, including text normalisation and cleaning. First, we discarded “stopwords” from the posts, which are words without inherent meaning, such as prepositions or articles. Next, we removed extraneous characters such as exclamation marks, asterisks, or emojis. Finally, we lemmatised the text, replacing verb forms with the subjunctive and used the TF-IDF model, which stands for “Term Frequency-Inverse Document Frequency,” to extract the most relevant words within each group (54). TF-IDF assesses the relative importance of a term in a group by comparing it with the rest, highlighting words that frequently recur in the texts of the same group and rarely appear in others. The terms with the highest TF-IDF values are considered the most relevant for that specific document. The topics were labeled by a group composed by clinicians and an engineer, who based their interpretation on the top 10 terms from the TF-IDF representation and by manually reviewing 25 representative tweets per category. The topics were labeled by a team composed of two clinicians (JCP and MAAM) and one engineer (FLA), who first reviewed independently the top 10 TF-IDF terms and a set of 25 representative tweets for each cluster. This initial step was followed by structured consensus meetings, where different interpretations were discussed until agreement was reached. This iterative consensus-based approach, commonly applied in qualitative research, was chosen to maximise the conceptual validity of the categories rather than statistical agreement. For transparency, the top 10 TF-IDF terms and 5 representative tweets used for labeling each cluster are provided in the Supplementary Materials (Supplementary Table S1).

Sentiment analysis was conducted analysing textual data to identify emotional undertones, categorising them. This approach allows for a nuanced understanding of the emotional landscape associated with discussions on psychotherapy. In the machine learning approach, we used a model trained to detect emotions to identify Ekman's six emotions: anger, disgust, fear, joy, sadness and surprise (55). The model used for the English tweets is English DistilRoBERTa-base (56), which achieves an accuracy of 66%. Regarding the Spanish-Tweets, we used the model called Robertuito (57) which achieves an accuracy of 55%. Both models have already been trained to classify the emotions of interest, so no additional training was necessary.

## 3 Results

### 3.1 Overall tweets

**Figure 1:**
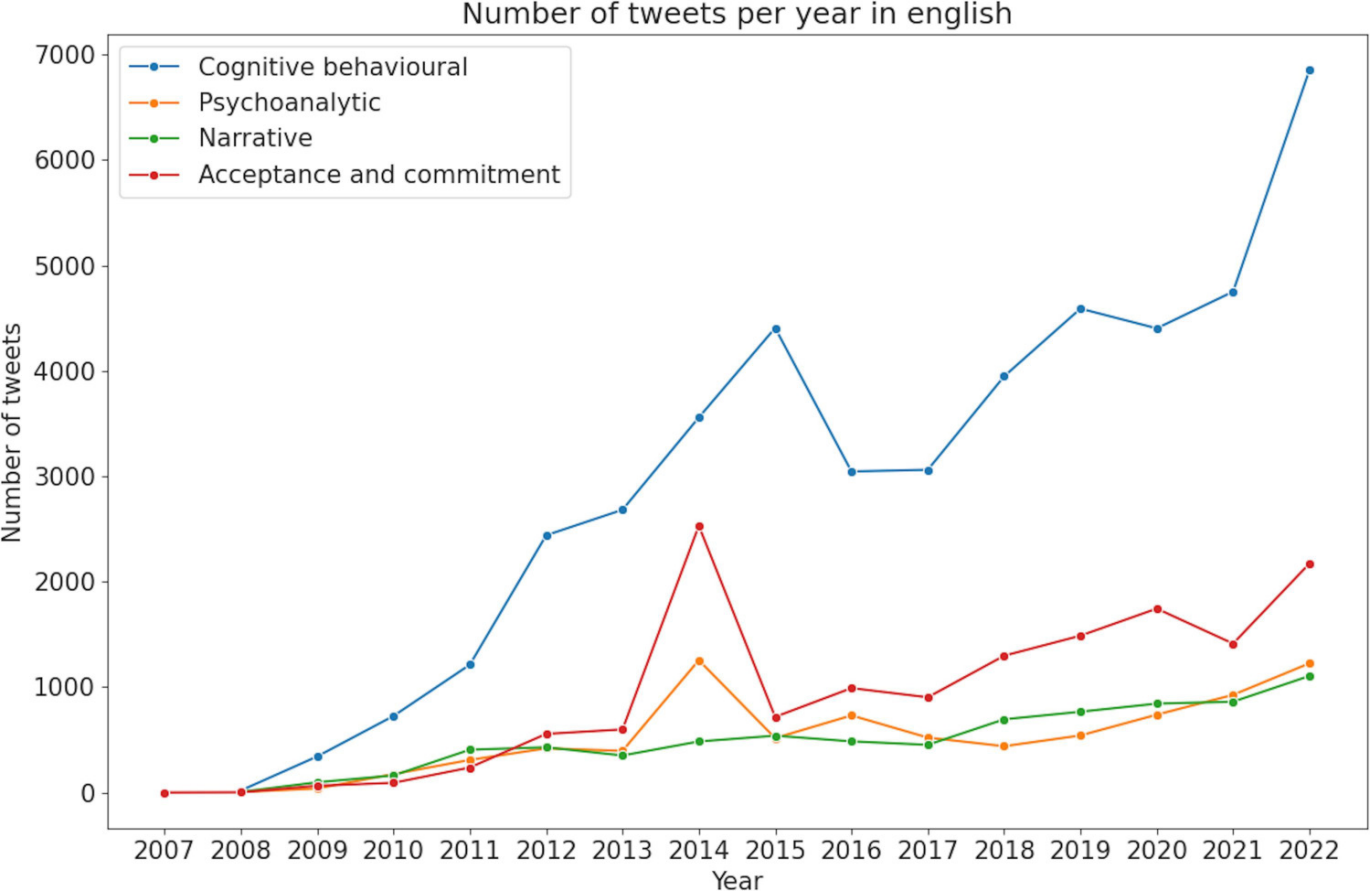
Number of tweets per year in English. Linear evolution of the number of tweets per year for each psychotherapy.

**Figure 2:**
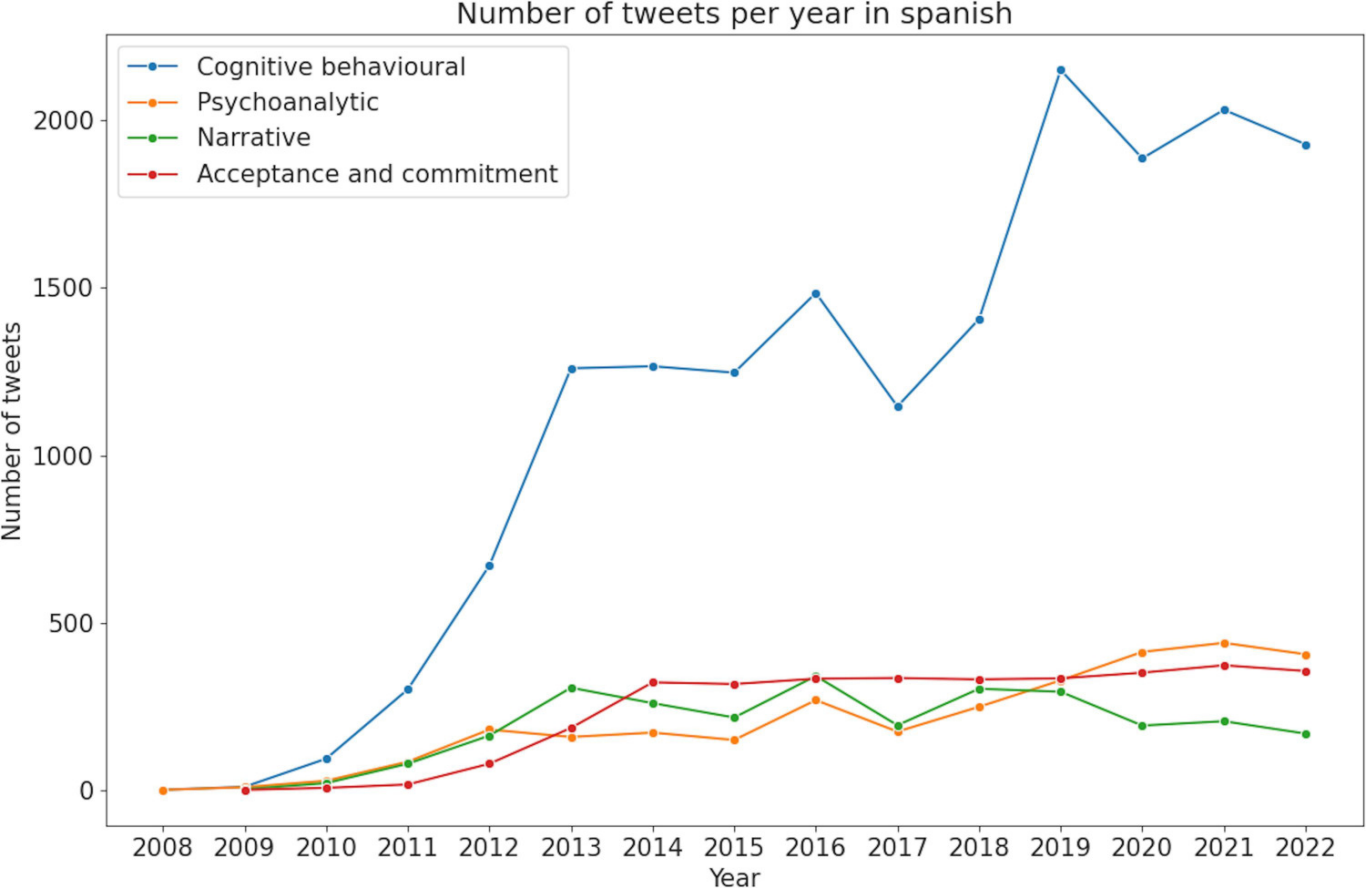
Number of tweets per year in Spanish. Linear evolution of the number of tweets per year for each psychotherapy.

A total of 102,946 tweets were retrieved for the selected psychotherapies. Tweets in English were more numerous (*n* = 76,878) compared to Spanish (*n* = 26,068). The type of psychotherapy that received the most publications was CBT, followed by ACT (Figures 1, 2).

### 3.2 Cognitive behavioural therapy

**Figure 3:**
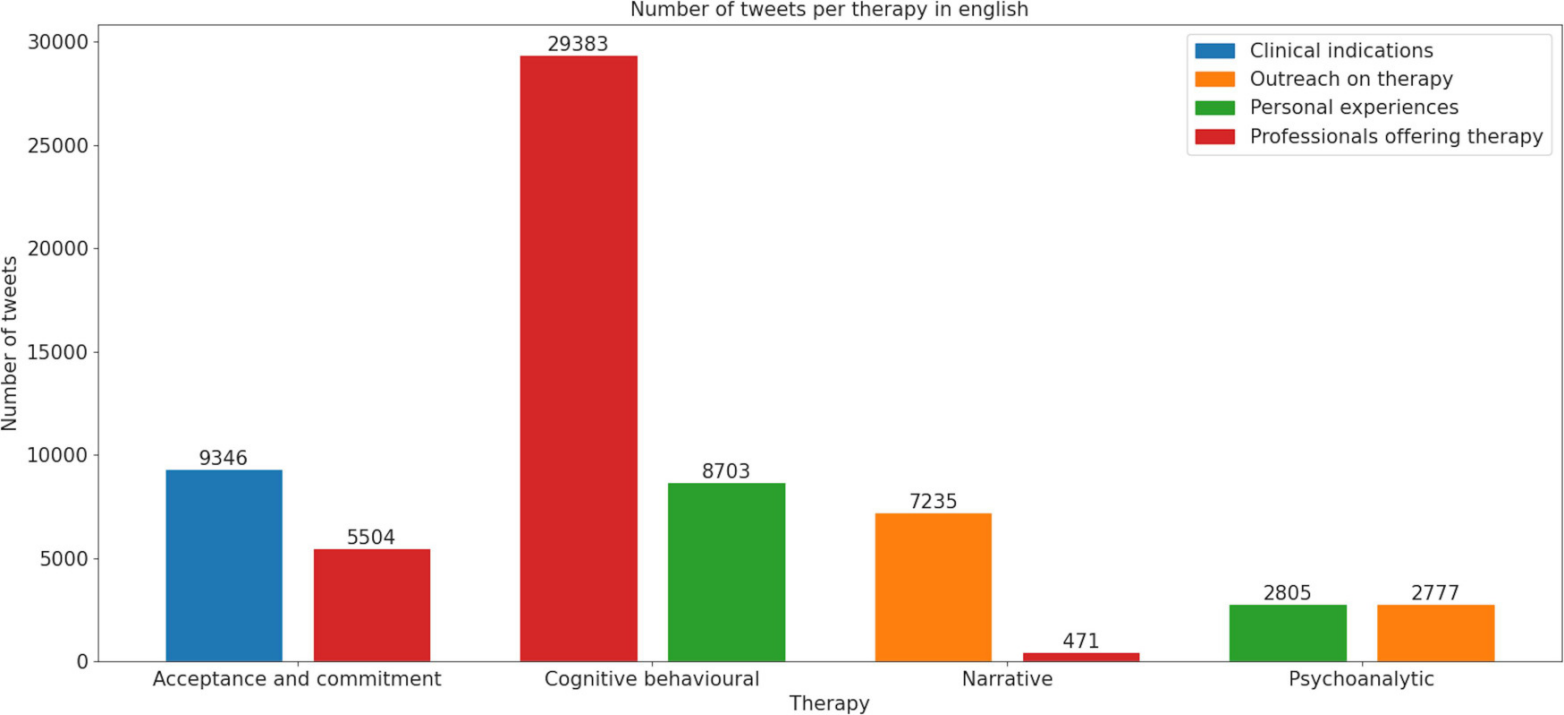
Number of tweets per therapy in English. The bars represent the two most frequent thematic category labels for each psychotherapy (ACT, CBT, NT, and PAT). The categories shown are: Clinical indications, Outreach on therapy, Personal experiences, and Professionals offering therapy. The y-axis indicates the number of tweets per category.

**Figure 4:**
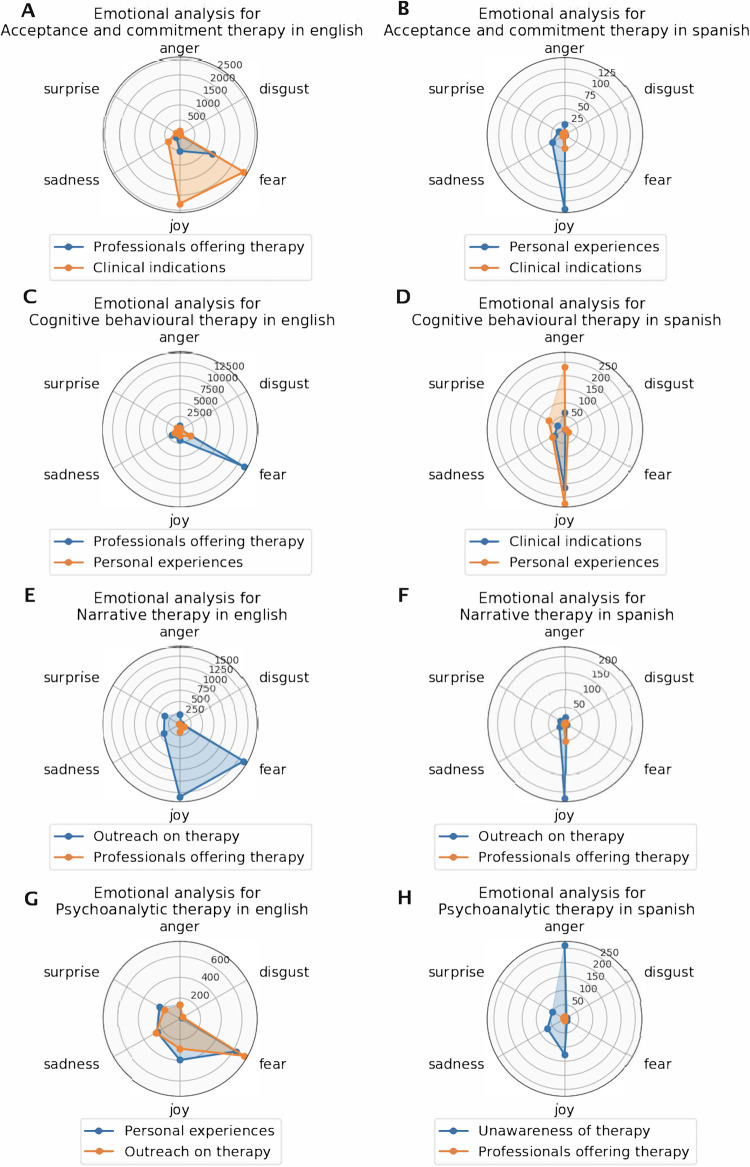
Emotional analysis for English and Spanish tweets. Sentiment analysis is shown for each psychotherapy in English (left) and Spanish (right): ACT **(A, B)**, CBT **(C, D)**, NT **(E, F)**, and PAT **(G, H)**. For each, the two most frequent topics are selected (differentiated by colours, orange and blue), reflecting their association with the primary sentiments (surprise, fear, joy, anger, disgust, sadness).

In the analysis of tweets about CBT in English, the primary focus was on professionals offering therapy and courses, with 29,383 tweets (Figure 3). This was followed by tweets discussing personal experiences (*n* = 8,703). A notable increase in tweets about therapy and courses has been observed since 2021 (Figure 1). Sentiment analysis showed that fear was the dominant emotion associated with CBT-related tweets, particularly those offering therapy and courses. This sentiment was also prevalent, though to a lesser extent, in tweets about personal experiences and clinical indications (Figure 4).

**Figure 5:**
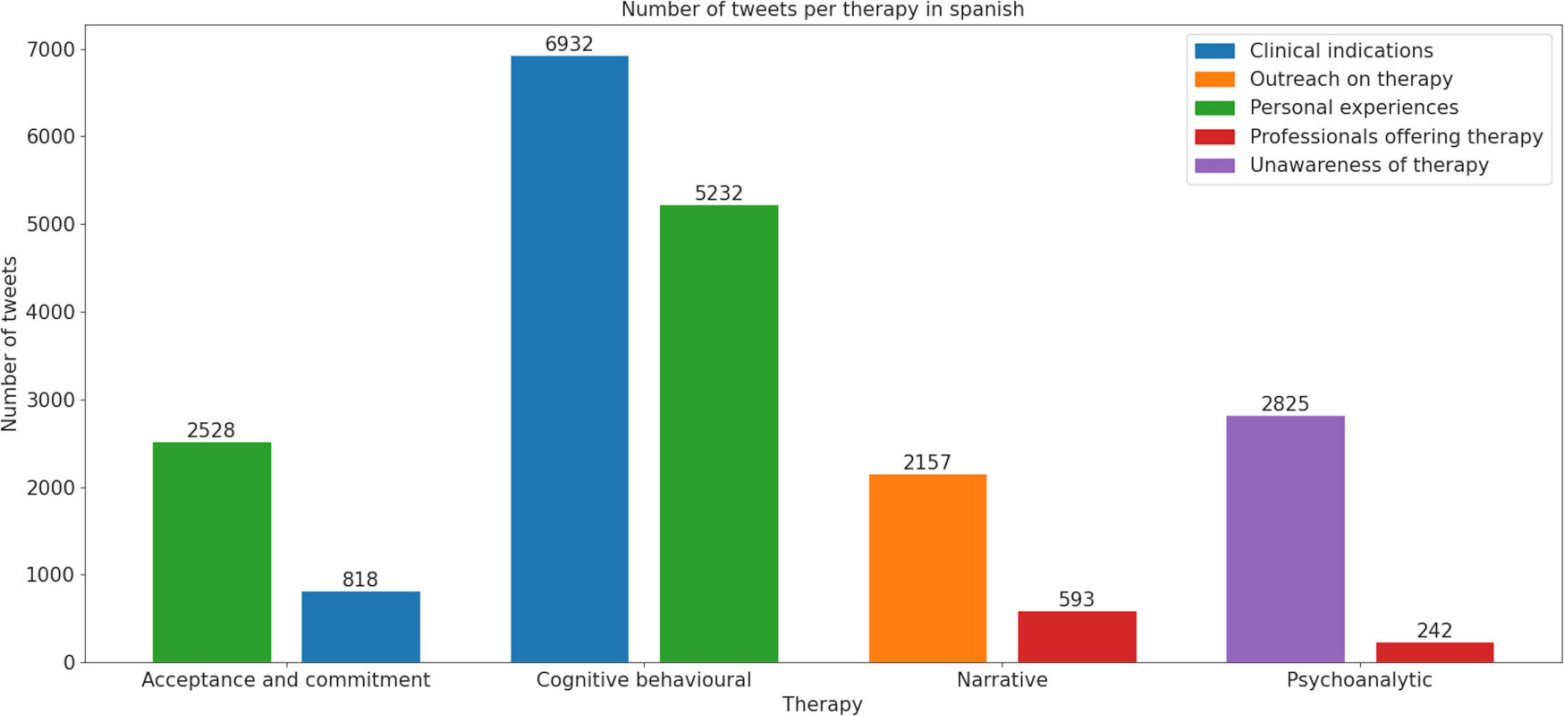
Number of tweets per therapy in Spanish. The bars represent the two most frequent thematic category labels for each psychotherapy (ACT, CBT, NT, and PAT). The categories shown are: Clinical indications, Outreach on therapy, Personal experiences, Professionals offering therapy, and Unawareness of therapy. The y-axis indicates the number of tweets per category.

For tweets in Spanish, the predominant topic was clinical indications with 6,932 tweets, followed by personal experiences (*n* = 5,232) (Figure 5). Tweets about CBT in general have shown a significant decline since 2019 (Figure 2). Sentiment analysis revealed that joy and anger were the predominant emotions in tweets about therapy experiences, with fewer expressions of surprise and sadness. Tweets concerning clinical aspects of CBT were mainly associated with joy (Figure 4).

### 3.3 Acceptance and commitment therapy

In the analysis of tweets about ACT in English, the majority focused on clinical indications, with a total of 9,346 tweets. Additionally, there were 5,504 tweets discussing courses and therapy offered by professionals (Figure 3). An increase in mentions of professionals offering therapy and courses starting from 2021 suggests a rise in the popularity or availability of professional training in ACT (Figure 1). Sentiment analysis indicated that fear was the predominant emotion associated with these tweets. Moreover, the presence of joy and sadness in tweets about clinical indications highlighted a complex emotional response associated with users' personal experiences with the therapy (Figure 4).

For tweets in Spanish, content analysis showed that personal experiences were the main topic, with 2,528 tweets, significantly surpassing those on clinical indications, with 818 tweets (Figure 5). Sentiment analysis revealed joy as the dominant emotion in personal experiences and clinical indications (Figure 4).

### 3.4 Psychoanalytic and psychodynamic therapy

The distribution of English tweets concerning PAT showed an even spread across three main topics. Personal experiences were the most discussed (*n* = 2,805 tweets), closely followed by outreach efforts on psychodynamic therapy (*n* = 2,777 tweets) (Figure 3). There was a significant peak in tweets concerning clinical indications in 2014, followed by a steady increase across all three topics, with a more pronounced rise in tweets about personal experiences since 2019 (Figure 1). Sentiment analysis indicated that fear was the predominant emotion across all topics, with sadness more associated with clinical discussions and joy primarily found in tweets about personal experiences and outreach efforts (Figure 4).

In Spanish, the content analysis revealed a predominant focus on the unfamiliarity of therapy (*n* = 2,825 tweets), with tweets from professionals offering therapy a distant second (*n* = 242 tweets) (Figure 5). Sentiment analysis showed that anger was the dominant emotion associated with the lack of awareness or ignorance about therapy, followed by lesser degrees of joy, sadness, and surprise (Figure 4).

### 3.5 Narrative therapy

The analysis of English tweets related to NT revealed that outreach was the predominant topic, with a significant total of 7,235 tweets, far surpassing the second most common topic of professionals offering therapy, which accounted for 471 tweets (Figure 3). Additionally, there has been a steady increase in the number of tweets over recent years (Figure 1). The primary emotions associated with these tweets were fear and joy (Figure 4).

In Spanish, the topic distribution was similar, with outreach again taking the lead (*n* = 2,157 tweets) and professionals offering therapy following (*n* = 593 tweets) (Figure 3). However, the trend in tweets about NT showed peaks and troughs, with a slight decline in interest from 2018 to 2019 onwards (Figure 2). Joy was the dominant emotion linked with posts about NT in both topics (Figure 4).

## 4 Discussion

Our study analysed tweets in English and Spanish related to four major psychotherapeutic approaches—ACT, CBT, PAT and NT—to identify prevailing themes, temporal trends, and associated emotional expressions. Using advanced natural language processing (BERTopic) and sentiment analysis models (DistilRoBERTa and Roberta-beto), we applied both qualitative and quantitative methods to characterise the online discourse surrounding these therapies on X. The results revealed variations in the emotional tone and thematic focus depending on the language and type of therapy, offering novel insights into how psychotherapy is publicly discussed and perceived on social media.

### 4.1 Why do psychotherapy discussions differ?

There are notable differences between the English-speaking and Spanish-speaking communities regarding the content analysis in tweets about therapies. Generally, clinical information and offers of courses and therapy by professionals dominate the tweets in English. In contrast, in the Spanish-speaking community, personal experiences are more common, as seen in discussions about ACT, or there is outright ignorance regarding certain therapies, such as PAT. Several factors might explain this phenomenon. Below, we outline several theoretical frameworks that may serve as hypothetical explanations for the observed patterns, particularly in relation to the sociocultural and systemic contexts in which these tweets may have been produced. These interpretations are exploratory and offered as a basis for generating future research hypotheses.

First, differences in the structure and access to mental health systems between English-speaking and Spanish-speaking countries could influence the nature of social media discourse. For example, in the United Kingdom where access to therapy and mental health education might be broader and more systematic (58–61), it is more likely that clinical and professional aspects are discussed. The situation in Spanish-speaking countries might be different. Latin American countries vary greatly in the accessibility and quality of health services provided, both between countries and across different socioeconomic conditions (62), often at the expense of mental health (63). Another factor to consider is the funding of the national health system. Countries like Great Britain and Spain follow the Beveridge model, financed by direct taxes, where the treated person does not pay for care. However, in regions such as the United States, where the healthcare system often operates on a for-profit basis (financed through insurance premiums, payroll taxes and tax deductions), there is a notable emphasis on marketing and promoting health services. This commercial aspect could explain the higher volume of tweets offering therapy services and professional courses in English-speaking countries (64). Such a market-driven approach to healthcare not only encourages providers to actively promote their services but also potentially increases the visibility and accessibility of such information through social media. This contrast is particularly striking when compared to many Latin American Spanish-speaking countries, where healthcare systems may lack universal health coverage, making inequities in access to quality healthcare and mental health services more apparent (62, 65).

Another possible factor to consider when evaluating differences between linguistic groups is how therapy is viewed by society, particularly regarding social stigma. While there are studies on the acceptance and integration of psychotherapy in different communities (66, 67), there are no previous studies that directly compare these differences between the two linguistic groups.

### 4.2 Impact of COVID-19: from crisis to opportunity

These thematic differences between English and Spanish tweets became more pronounced starting in 2020. Since then, the number of tweets in English offering treatment and courses, and promoting therapy, has steadily increased. Specifically, CBT and ACT have seen a rise in tweets offering therapies and courses, with a notable increase since 2021. Similarly, therapies like PAT and NT have seen an uptick in tweets with informational content, aimed at efforts to promote or spread information about the therapy. One possible explanation of this finding might be the coincidence with the COVID-19 pandemic, which heightened attention to mental health issues and increased the need for accessible mental health services (6, 8, 68, 69). Similarly, the increase in digital outreach and online resources (15, 16), helped disseminate information about these therapies more broadly. There is also prior evidence on the adaptability of various types of therapy to online formats, making them particularly suited to remote delivery and capable of effectively treating various conditions (70–72). Spanish-language tweets did not show a comparable increase in promotional content during this period. This divergence may reflect differences in the digital communication strategies employed by professionals, or in the way psychotherapy is publicly discussed across linguistic communities. Given the lack of geolocation data, further research would be needed to explore whether systemic factors such as healthcare infrastructure, mental health literacy, or stigma play a role in shaping these patterns.

### 4.3 Therapy vibes: emotional responses and informational needs

Sentiment analysis also revealed differences between tweets in English and Spanish. The primary emotion in English-speaking tweets—associated with clinical information and offers of therapy and courses—was fear, possibly reflecting concerns or uncertainties about managing clinical conditions, or suggesting a general atmosphere of apprehension related to aspects of the therapy (CBT, for example). This contrasts with Spanish-speaking tweets, such as those related to ACT, where there is an association between tweets about personal experiences and the feeling of joy, suggesting a positive reception and potentially effective valuation of the therapy among Spanish-speaking users. Additionally, a significant presence of anger was noted in Spanish tweets related to PAT, particularly linked to a lack of understanding about these therapies. This predominance of anger may reflect frustration or conflicts over the perception of these therapeutic practices and indicate a broader accessibility issue (11). Often, these conversations in Spanish pivot towards personal experiences or highlight a notable deficiency of information about available therapies, further underscoring the challenges in therapy recognition and accessibility.

### 4.4 Implications

The findings of this study have significant implications for understanding the global discourse on psychotherapy and the varying perceptions across different linguistic and cultural communities. The differing themes and emotional tones in tweets highlight the need for culturally tailored mental health communication strategies and social media-based approaches. Healthcare providers and policymakers should consider these differences when designing mental health interventions and public health campaigns (59, 73). The prominence of fear in English-speaking tweets suggests a need for addressing apprehensions and uncertainties about therapy through clear and accessible information. Conversely, the positive sentiment associated with personal experiences in Spanish-speaking tweets indicates the potential effectiveness of leveraging personal testimonials in mental health advocacy. Additionally, the significant presence of anger related to ignorance in Spanish-speaking communities underscores the urgent need for improved mental health literacy and accessibility in these regions. Moreover, the emotional climate in each language group may influence the effectiveness of promoting psychotherapies. In Spanish-speaking communities, where anger is predominant in PAT tweets, this emotional barrier could make it more challenging for professionals to engage in outreach, potentially perpetuating the cycle of limited understanding. In contrast, the fear prevalent in English-speaking tweets might be more effectively addressed through reassurance and clear information.

This study also underscores the value and practicality of using X and similar social media platforms for population-level research*.*The vast amount of real-time data on these platforms provides unique insights into public opinions and behaviours. Social media analysis allows a dynamic understanding of how mental health topics evolve over time and across different cultures and languages. This innovative approach complements traditional research methods, offering a broader and more immediate perspective on public health issues, and underscores the potential of digital platforms to inform and enhance mental health strategies globally.

### 4.5 Strengths and limitations

This study has several limitations that must be considered. One significant limitation is the reliance on X data, which may not fully capture the broader public sentiment. The platform's user base might not be representative of the general population, as it tends to skew younger, more urban, and tech-savvy.

Furthermore, the study does not consider regional differences within English-speaking and Spanish-speaking communities, which could affect the generalisability of the results. For instance, in English-speaking countries, access to mental health services and attitudes toward psychotherapy can vary considerably. In the UK, there is greater familiarity with public therapy programmes and accessible mental health services (74), whereas in the United States, psychotherapy is more commercialised, and access often depends on socioeconomic status (75). There are also sociocultural differences within specific regions or countries not addressed in this work. The perception of psychotherapy may differ significantly between urban and rural areas or across different social classes and ethnic communities (76). In some areas, especially rural regions or contexts where mental health services are not easily accessible, psychotherapy may be perceived with stigma or seen as an elitist service (77, 78). It is also important to note that our dataset does not include geolocation information; all analyses are based solely on language (English or Spanish). As such, any references to healthcare systems or country-specific factors in the discussion are intended as hypothetical interpretations and should not be construed as data-driven conclusions.

Moreover, the brevity and character limits of tweets may restrict the depth of information shared, potentially oversimplifying complex mental health experiences and treatments. The analysis also primarily captures the publicly available tweets, potentially overlooking private or protected conversations that could provide additional insights. To address these limitations, future research should aim to include a more diverse range of social media platforms and consider regional variations to provide a more comprehensive understanding of public perceptions of psychotherapy. Additionally, longitudinal studies incorporating sociodemographic variables such as location, age, and sex could offer deeper insights into how these factors influence attitudes towards psychotherapy over time.

Another potential limitation is the accuracy of the emotion detection models used in the analysis. These models were trained in an unsupervised manner, which, while useful for detecting general emotional undertones, does not always capture the full complexity and nuance of emotions in social media discourse. Future studies should refine emotion detection models, particularly for languages and contexts where accuracy may be lower. Moreover, incorporating human validation of the model's outputs could further enhance the robustness of the emotional analysis.

A significant restriction of this study is the deliberate exclusion of certain approaches (e.g., mindfulness), despite their relevance in public discourse, due to the significant amount of non-psychotherapeutic content they generate in online discussions. Future research could broaden the scope to include other therapies for a more comprehensive analysis.

A further limitation is that we did not apply automated bot-detection techniques. While most tweets appeared to reflect human-generated discourse (79), it is possible that some automated or promotional content remained in the dataset. Future research should consider integrating bot-filtering methods to enhance data accuracy.

This study presents several notable strengths. By combining NLP techniques with both qualitative and quantitative methods, we offer a comprehensive analysis of how psychotherapy is discussed on social media. The use of BERTopic for unsupervised topic modelling and transformer-based models for emotion detection allows for nuanced insight into the themes and emotional tone of tweets over time. A key strength of this work is the inclusion of tweets in both English and Spanish, which enables a novel cross-linguistic comparison rarely addressed in prior studies. This bilingual design broadens the generalisability of the findings and captures language-specific patterns in public discourse, revealing differences in how psychotherapies are perceived, described, and emotionally evaluated depending on the language of communication. The inclusion of both languages also enhances the practical relevance of the study for global digital mental health strategies. Furthermore, the 15-year time frame and the focus on current issues—such as the impact of the COVID-19 pandemic and the growing role of digital platforms in mental health awareness—add further relevance to contemporary discussions in mental health research and policy. Together, these elements contribute to a robust and original understanding of public attitudes toward psychotherapy and offer valuable insights for healthcare providers, researchers, and policymakers aiming to design effective and inclusive communication strategies.

## 5 Conclusion

This study sheds light on the diverse ways in which psychotherapies are perceived and discussed across English-speaking and Spanish-speaking communities on X over a 15-year period. By analysing tweets about various psychotherapies, including ACT, CBT, PAT, and NT, we identified significant differences in thematic concerns, emotional tones, and attitudes between the two linguistic groups. English-speaking tweets predominantly focus on clinical information and therapy offers, while Spanish-speaking tweets are more centered around personal experiences. Notably, Spanish tweets about PAT were marked by significant anger, reflecting frustration and lack of understanding, whereas English tweets were primarily characterized by fear, particularly in relation to clinical services and therapies like CBT. Additionally, the emotional climate differed, with joy emerging as a dominant feeling in Spanish tweets related to ACT, contrasting with the more apprehensive tone in English tweets. The impact of COVID-19 is also evident, as the volume of tweets offering therapy services, surged starting in 2021, signaling the pandemic's role in increasing the public's attention to mental health services.

By understanding these nuances, healthcare providers, policymakers, and mental health advocates can develop more effective, culturally sensitive approaches to promoting mental health consciousness and accessibility. This study underscores the importance of considering linguistic and cultural contexts in global mental health efforts and highlights the potential of social media as a valuable tool for gaining insights into public perceptions of psychotherapy.

## Data Availability

The original contributions presented in the study are included in the article/Supplementary Material, further inquiries can be directed to the corresponding author.
