# Supplementary material for: Public perceptions and emotional trends of psychotherapy: a 15-year analysis across English and Spanish language communities on X (twitter)
Source: Front Digit Health. 2025 Dec 16;7:1598237. doi: 10.3389/fdgth.2025.1598237 (PMC12748249; doi:10.3389/fdgth.2025.1598237)
Supplement: Supplementary file 1 [file Datasheet1.pdf]

Supplementary Material

Section 1: Keywords used for tweets research:

1. **Keywords for Psychoanalysis**

- English keywords: "psychoanalysis", "psychoanalytic psychotherapy", "psychodynamic psychotherapy", "brief psychodynamic psychotherapy", "transference-focused psychotherapy", "psychodynamic therapy", "analytic psychotherapy", "psychodynamic treatment", "psychoanalytic theory", "Freudian therapy", "object relations therapy", "transference-focused therapy".

- Spanish keywords: "psicoanálisis", "psicoterapia psicoanalítica", "psicoterapia psicodinámica", "psicoterapia psicodinámica breve", "psicoterapia focalizada en la transferencia", "terapia psicodinámica", "psicoterapia analítica", "tratamiento psicodinámico", "teoría psicoanalítica", "terapia freudiana", "terapia de relaciones objetales", "terapia focalizada en la transferencia".

2. **Keywords for Acceptance Commitment therapy**

- English keywords: "acceptance and commitment therapy", "ACT", "AC therapy", "acceptance therapy", "commitment therapy", "acceptance-based therapy", "ACT for stress", "ACT mental health", “acceptance commitment treatment”, acceptance commitment”.

- Spanish keywords: "terapia de aceptación y compromiso", "TAC", "terapia AC", "terapia de aceptación", "terapia de compromiso", "terapia basada en aceptación", "ACT para el estrés", "ACT salud mental", "tratamiento de aceptación y compromiso", "aceptación y compromiso".

3. **Keywords for Cognitive behavioral therapy**

- English keywords: "cognitive behavioral therapy", "CBT", "cognitive therapy", "behavioral therapy", "cognitive restructuring", "CBT techniques", "CBT mental health".

- Spanish keywords: "terapia cognitivo-conductual", "TCC", "terapia cognitiva", "terapia conductual", "reestructuración cognitiva", "técnicas de TCC", "TCC salud mental".

4. **Keywords for Narrative therapy**

- English keywords: "narrative therapy", "storytelling therapy", "narrative-based therapy", "therapeutic narrative", "narrative techniques", "narrative approach", "narrative counseling", “narrative psychology”.

- Spanish keywords: "terapia narrativa", "terapia de cuentos", "terapia basada en narrativas", "narrativa terapéutica", "técnicas narrativas", "enfoque narrativo", "psicología narrativa".

frontiers

Table 1: Examples of tweets related to different psychotherapeutic approaches, organized by therapy type, topic, language, representative anonymized tweets, and the ten most representative TF-IDF terms. Tweets were anonymized and edited for clarity while preserving the original meaning. Labels for the topics were derived from a combination of automated clustering (BERTopic, c-TF-IDF) and consensus interpretation by the research team.

Therapy	Main Topics	Language	Representative Tweet (anonymized example)	10 TF-IDF Terms
**Cognitive Behavioural Therapy**	Professionals offering therapy and courses	English	1. *Cognitive-behavioural therapy (CBT) can help people cope with everyday challenges by working on changing thoughts and behaviours. A free 12-week online program is available.*<br>2. *Please do not silently struggle with mental health conditions such as anxiety or depression, ThinkingThrough is here to support you with our professional, qualified and expert therapists with one to one online CBT sessions. Fill in our contact form today*<br>3. *Free online therapy now available in the Western Isles * Private 1-to-1 sessions with qualified CBT therapist * no need to see a GP * no video calling, just typing * flexible appointments to suit you.Enhance your mental well-being with practical tools and strategies. We provide fast, free access to cognitive behavioural therapy and related resources. Learn more and sign up now.*<br>4. *Sharing this wonderful opportunity for 6 free, CBT groupwork sessions on behalf of the lovely lady that is…*<br>5. *NHS Fife's Psychology Service runs a 2-hour online class that applies CBT principles to sleep, covering healthy routines and strategies for better rest*	CBT, therapy, sessions, online, program, course, free, support, psychology, skills

**Cognitive Behavioural Therapy**	Personal experiences	English	1. *This year has been really tough. I just started cognitive behavioural therapy recently and have now completed two sessions. Being able to talk about things feels supportive, and I'm hopeful it will make coping with daily challenges easier.*<br>2. *When CBT was first recommended to me, my first reaction was: how could that possibly help with my anxiety and all the issues tied to living with a uterus? It definitely says a lot about where my mindset was back then.*<br>3. *For me, mindfulness informed by CBT is what I use to make space for myself whenever possible. I also rely on focus settings on my phone to help me stay centred and relaxed.*<br>4. *Funny you're into that, Bekk! My own experience with CBT wasn't very effective, but I'm genuinely glad it's working well for you.*<br>5. *Since mindfulness plays such a big role in my life, I often find it more useful in different contexts than CBT. That said, CBT was incredibly effective in helping me overcome a severe fear of heights.*	CBT, anxiety, mindfulness, life, struggle, experience, coping, support, mindset, growth
**Cognitive Behavioural Therapy**	Clinical indications	Spanish	1. *La terapia cognitivo-conductual (TCC) ha demostrado eficacia en el manejo del insomnio en personas que viven con dolor crónico.*<br>2. *La TCC para la psicosis (TCCp) puede ser eficaz para reducir tanto el riesgo de transición a la psicosis como la gravedad de los síntomas psicóticos atenuados durante la fase prodrómica.*<br>3. *Investigaciones de XXX destacan que la TCC grupal es una opción terapéutica valiosa para el insomnio, con la colaboración de los departamentos de Neurofisiología, Medicina Preventiva y Psiquiatría.*<br>4. *La TCC es muy eficaz para abordar los miedos y las inseguridades. A diferencia del coaching, este tipo de apoyo debe ser proporcionado por psicólogos formados. Podría resultarte útil leer más al respecto.*	Terapia cognitivo-conductual, insomnio, crónico, dolor, síntomas, miedos, eficaz, intervención, depresión, ansiedad.

			5. *Un panel de expertos de la Sociedad Española de Psicología Clínica y de la Salud concluye que la TCC es una intervención eficaz para el dolor crónico.*	
**Cognitive Behavioural Therapy (CBT)**	Personal experiences	Spanish	1. *La ansiedad y la depresión pueden ser provocadas por factores inesperados, especialmente cuando las emociones se acumulan con el tiempo. La terapia cognitivo-conductual (TCC) cambió mi vida al ayudarme a afrontar el tipo específico de ansiedad que tenía. Este libro también me dio una comprensión más clara — ¡altamente recomendado!*<br>2. *Pasé cuatro años en terapia, pero el miedo realmente nunca desapareció hasta que tomé un curso de terapia cognitivo-conductual (TCC) de seis meses, que me ayudó más que todos esos años anteriores. Incluso dejé atrás el psicoanálisis y mis estudios de psicología.*<br>3. *Solía tener ataques de pánico frecuentes y tomé antidepresivos por un tiempo, pero lo que realmente más me ayudó fue la terapia cognitivo-conductual (TCC).*<br>4. *Probé la terapia cognitivo-conductual (TCC) pero no la encontré útil. Pensé en cambiarme al psicoanálisis, pero ha sido criticado tan duramente y etiquetado como anticuado que ahora no estoy seguro de qué hacer con mi vida. Me he dado un año más.*<br>5. *Hola XXXX, sí — en mis días más oscuros recordaba cuando escribía en Puebla. Durante la terapia cognitivo-conductual (TCC) practiqué escribir pensamientos negativos para sentir alivio; ese ejercicio me ayudó a motivarme y eventualmente me inspiró a convertirlo en un libro.*	Terapia cognitivo-conductual, ansiedad, depresión, terapia, miedo, pánico, antidepresivos, pensamientos, vida, terapia.
**Acceptance and Commitment Therapy**	Clinical indications	English	1. *One of our members has co-authored a recently published review titled "What is the evidence for self-help Acceptance and Commitment Therapy? A systematic review and meta-analysis."*	ACT, depression, pain, impulsivity, headache,

			2. *Study exploring the effectiveness, cost-effectiveness, and physiological outcomes of Acceptance and Commitment Therapy (ACT) and Behavioural Activation in relation to low back pain and depression (#IMPACTstudy).*<br>3. *Honoured to be referenced in a paper examining the benefits of ACT, particularly for Muslim populations, framed within the Ecological Systems Theory perspective.*<br>4. *Symposium presentation WC17: examining the impact of ACT on addiction severity and impulsivity among people who use methamphetamine.*<br>5. *Our latest publication reports on a randomized controlled trial testing Acceptance and Commitment Therapy for individuals with primary headache disorders.*	outcomes,effectiveness, study, trial, efficacy.
**Acceptance and Commitment Therapy**	Courses and therapy offered by professionals	English	1. *"Learning ACT has really strengthened my therapeutic skills and confidence." Great to receive such feedback from participants in our Intermediate Acceptance and Commitment Therapy training. The next six-session programme begins on 4 November 2019 — details available.*<br>2. *This Acceptance and Commitment Therapy training in Lincoln could be of interest to PPA members. Reduced registration fees are available until 3 February.*<br>3. *New online programme in ACT now open for registration — get your ticket! #ACT*<br>4. *Do you support people living with chronic pain or long-term conditions? Take part in our two-day ACT training on 27–28 March in XXXX. Includes a free ACT book and the chance to enjoy the nearby nature reserve during lunch breaks.*<br>5. *Exciting news — a first look at his debut online course on Acceptance and Commitment Therapy has just been shared. Don't miss it!*	ACT, training, course, programme, online, registration, skills, confidence, sessions, ticket

**Acceptance and Commitment Therapy**	Personal experiences	Spanish	1. *¿Alguna vez has investigado la Terapia de Aceptación y Compromiso? Me ayudó mucho a manejar las dificultades relacionadas con el trastorno límite de la personalidad.*<br>2. *Estoy muy orgulloso de un amigo que está superando una mala racha y, sin siquiera darse cuenta, ha estado utilizando técnicas de la Terapia de Aceptación y Compromiso. Yo no tuve nada que ver con eso, pero es genial ver cómo la psicología marca la diferencia.*<br>3. *Hace un par de años descubrí la Terapia de Aceptación y Compromiso, y las herramientas que me proporcionó han sido muy útiles. Mi relación con la ira ha mejorado mucho, aunque todavía queda camino por recorrer — como en cualquier relación, hay altibajos.*<br>4. *Intentar aplicar la Terapia de Aceptación y Compromiso a uno mismo no siempre funciona — la auto-terapia tiene sus límites, incluso cuando insistes en hacerlo.*<br>5. *La Terapia de Aceptación y Compromiso realmente me devolvió la vida, especialmente durante la pandemia. Vale la pena apoyarse en enfoques con evidencia sólida en lugar de en aquellos que ya están desactualizados.*	Vida, terapia, ansiedad, depresión, amigo, enfado, pensamientos, habilidades, experiencia.
**Acceptance and Commitment Therapy**	Clinical indications	Spanish	1. *Recursos clínicos: materiales de audio diseñados para trabajar la autocrítica y la vergüenza mediante la Terapia de Aceptación y Compromiso.*<br>2. *Ensayo clínico exploratorio que evalúa la Terapia de Aceptación y Compromiso como complemento de la terapia de relajación psicoeducativa en personas con dolor crónico.*<br>3. *#SALUDMENTAL: La desregulación emocional es una característica central del #TLP, lo que la convierte en un objetivo clave en las intervenciones basadas en la evidencia, incluida la Terapia de Aceptación y Compromiso.*<br>4. *"Experiencia con un programa de psicoeducación multifamiliar en patología dual (DI + TUS): estudio piloto" junto con "Intervención grupal de Terapia de Aceptación y*	Depresión, TLP, TOC, dolor, ensayo, mindfulness,psicoterapia, intervención, vergüenza.

			Compromiso para el trastorno obsesivo-compulsivo resistente al tratamiento."<br>5. *Terapia cognitiva basada en mindfulness, Terapia de Aceptación y Compromiso y psicoterapia positiva en el tratamiento de la depresión mayor.*	
**Psychoanalytic and Psychodynamic Therapy**	Personal experiences	English	1. *Seems like my therapist is still recovering after reading the dozen short stories I wrote as part of my therapy. Maybe I gave them a little too much of a window into my mind. My conclusion? They're burnt out — might be time to find someone new.*<br>2. *To break free from those "unconscious patterns," I found psychoanalysis incredibly valuable. I enjoy it because it feels scientific in its exploration of emotions and avoids being dogmatic.*<br>3. *Life feels positive now. I'm glad not to let depression define me anymore. After 15 years of psychoanalytic treatment, I've managed to move beyond painful traumas. Through prayer and therapy, I've learned to love myself more than ever.*<br>4. *I've grown through psychoanalysis, navigating depression, facing anxiety, working through shame, exploring AEDP, and using the Change Triangle. What has helped you grow?*<br>5. *My therapist interrupted me mid-post, but here's the white background version anyway. Psychoanalysis won't cure my condition, so you'll keep seeing this kind of content from me.*	psychoanalysis, therapist, life, depression, anxiety, trauma, growth, shame, childhood, therapy
**Psychoanalytic and Psychodynamic Therapy**	Outreach efforts on psychodynamic therapy	English	1. *Expand your knowledge of the role relationships play in mental health through this 8-session online programme led by psychoanalyst Marcus Evans and colleagues.*<br>2. *"Psychoanalysis and Ethics" by David Black, part of The New Library of Psychoanalysis, is a key text for anyone interested in the role of ethics in psychoanalytic thought and practice.*	psychoanalysis, programme, ethics, relationships, psychotherapy, theory, practice, mind, existence, Lacan

			3. *A core idea in psychoanalysis is that the mind is inseparable from the body — that being human is to be anima.*<br>4. *What should we expect when beginning psychoanalytic psychotherapy or full psychoanalysis? The two approaches are often debated, but since they share similar aims and foundations, I'll leave that discussion aside for now. (1/2)*<br>5. *According to Lacan, psychoanalysis is not primarily a method for treating mental symptoms, but rather a practice that confronts us with the deepest aspects of human existence — not guiding us to adjust, but to face that radical dimension.*	
**Psychoanalytic and Psychodynamic Therapy**	Clinical indications	English	1. *Research on the effectiveness of psychoanalysis shows that, overall, it performs as well as many standard medical treatments — with success rates of around 80%.*<br>2. *Evidence on long-term psychodynamic therapy (LTPP) has been mixed, but two recent meta-analyses indicate positive outcomes. In our own analysis, recovery rates for LTPP were comparable to those seen in control conditions.*<br>3. *PET imaging demonstrates how psychodynamic therapy for depression may bring about functional changes in the brain.*<br>4. *This Wednesday, a BPA psychoanalyst will reflect on the role psychoanalysis in depression, drawing on Freud's Mourning and Melancholia and its continuing relevance for understanding patients' struggles.*<br>5. *In a major trial of depression, psychodynamic therapy proved as effective as CBT, and even showed higher rates of remission and improved well-being*	psychoanalysis, borderline, depression, effectiveness, therapy, remission, outcomes, personality,treatment, trial
**Psychoanalytic and Psychodynamic Therapy**	Unfamiliarity of therapy	Spanish	1. *Aparece el psicoanálisis… hora de irme a dormir.*<br>2. *Ahora mi feed está lleno de gente diciendo que si no confiamos en el psicoanálisis porque no entendemos sus principios, entonces tampoco deberíamos confiar en la*	Psicoanálisis, qué, estudiando, principios, confianza, medicación, tarot, café, cama, feed

			medicación. Y yo, simplemente aquí sentado con palomitas viendo cómo se desarrolla todo 😂.<br>3. *Primer día de estudio, y por supuesto empieza con psicoanálisis. Eso solo puede significar una cosa: café.*<br>4. *Ayer en mi clase de psicoanálisis, estaba medio autoanalizándome mientras sacaba cartas del tarot… y estas fueron las que salieron:*<br>5. *Todo el mundo cree saber qué es el psicoanálisis — excepto los propios psicoanalistas… porque si realmente lo supiéramos, no existiría la contingencia.*	
**Psychoanalytic and Psychodynamic Therapy**	Professionals offering therapy and courses.	Spanish	1. *Mañana realizaremos la segunda sesión del curso de psicoanálisis. Para aprovecharlo al máximo, sugerimos ver El inquilino (Polanski, 1976). Inscríbete enviando tu nombre completo por correo electrónico.*<br>2. *Un colega está ofreciendo un curso de psicoanálisis — asequible y con un programa sólido. Hoy hay una charla por Zoom sobre el curso, ¡no te la pierdas!*<br>3. *El Comité de Psicoanálisis de Niños y Adolescentes del Instituto APM invita al "Curso de Psicoanálisis con Niños y Adolescentes," dirigido a psicoanalistas y estudiantes a partir del séptimo semestre. Inscríbete aquí: https://bit.ly/CURSO23 #Psicoanálisis*<br>4. *NUEVO CURSO GRATUITO DE PSICOANÁLISIS en XXXXX: El malestar en la cultura.*<br>5. *Final spots available for the psychoanalysis course on the individual and society at our centre. Interested? Don't miss your chance.*	Psicoanálisis, curso, sesión, programa, inscribirse, comité, gratuito, centro, formación, seminario web.
**Narrative Therapy**	Professionals offering therapy and courses.	English	1. *I'm running a trauma-focused RE/CBT group that integrates key elements from CPT Trauma Narrative therapy along with my own ICT approach. Taking place in XXX, it's aimed at those affected by early adversity or recent trauma.*	narrative, therapy, sessions, online, certified, workshop,

			2. *Join us in XXXX for a Narrative Therapy group workshop. Through a creative exercise on values, beliefs, and support systems, we'll connect these insights to everyday life in a lasting way.*<br>3. *Our 5-day Narrative Therapy Certificate Program begins today! Grateful to all participants for choosing us for their professional development.*<br>4. *Day one of our 5-day Narrative Therapy webinar series is here! Excited to see everyone logging in to learn how to apply this model in practice.*<br>5. *Wrapping up day 2 of our Level 2 Narrative Therapy training online with a fantastic, engaged group. Looking forward to continuing in March*	webinar, join, trauma, beliefs.
**Narrative Therapy**	Outreach	English	1. *Curious about narrative therapy? Here's a clear and engaging introduction — beautifully written, easy to follow, and full of profound insights. Not widely known in the UK, but definitely worth discovering. #NarrativeTherapy*<br>2. *Looking for a simple overview of narrative therapy? This introduction makes the concepts easy to understand and accessible.*<br>3. *Narrative therapy helps create distance between the person and the problem, encouraging people to view difficulties as external rather than part of their identity.*<br>4. *"Narrative therapy allows us to tell a deeper truth: something happened to me, but I am not defined by what happened."*<br>5. *With narrative therapy, the problem is seen as separate from the individual. This approach empowers people to use their own strengths to reduce the impact of challenges in their lives.*	narrative, therapy, stories, life, tell, Epston, importance, trauma, access, treatment.

**Narrative Therapy**	Outreach	Spanish	1. *La terapia narrativa es una herramienta valiosa para trabajar el duelo. Encantado de haber asistido a los Premios de Microrrelatos XXX 2018.*<br>2. *Una idea en la terapia narrativa es reformular la manera en que nos relacionamos con nuestros problemas, creando cierta distancia entre nosotros y las dificultades que enfrentamos.*<br>3. *La terapia narrativa se considera uno de los modelos clave dentro de las terapias breves. Se centra en la relación de la persona con su entorno, viendo a los seres humanos como narradores que construyen significado a través de sus historias.*<br>4. *Para la terapia narrativa, es esencial deconstruir las historias saturadas de problemas y luego transformarlas utilizando técnicas como la "externalización", la "reautorización" y las "contranarrativas".*<br>5. *El objetivo de la terapia narrativa es enriquecer la historia de una persona incorporando detalles pasados por alto y ensombrecidos por el problema — co-creando juntos una narrativa alternativa.*	Narrativa, terapia, duelo, objetivo, historias, entorno, narradores, externalización, reautorización, alternativa.
**Narrative Therapy**	Professionals offering therapy and courses.	Spanish	1. *Vamos a deconstruir historias en 2016 — nuestro curso de Terapia Narrativa comienza este enero.*<br>2. *Oportunidades de formación de AETEN (Asociación Española de Terapia Narrativa): Máster en Terapia Narrativa y Trabajo Comunitario. Más información: https://aeten.es/formaciones/*<br>3. *La sesión de hoy del curso de Terapia Narrativa comienza a las 18:00 — con supervisión en vivo incluida.*<br>4. *¡Solo queda una semana para que comience el curso de Terapia Narrativa! Más información en…*<br>5. *Descentrar el papel del terapeuta es un acto político. Únete a nuestra formación online personalizada en Terapia Narrativa — diseña tu propio curso y obtén tu…*	Narrativa, terapia, curso, formación, sesión, supervisión, asociación, en línea, personalizada, máster.

Section 2: Illustrative Examples of Emotion-Related Tweets by Topic

To provide a more concrete understanding of the emotional patterns identified in our computational analysis, we present below several anonymised example tweets representative of the most relevant thematic and emotional categories that are discussed on discussion.

1. **Fear associated with clinical information and offers of therapy or courses (English)**

- *"It really upsets me when I hear that someone has spent their time and money on poorly delivered cognitive-behavioral therapy. Psychotherapy often feels vague and confusing, which doesn't help. That's why I put together this guide showing what good and bad CBT usually look like."*

2. **Fear associated with personal experiences with CBT (English):**

- *When CBT was first recommended to me, my first reaction was: how could that possibly help with my anxiety and all the issues tied to living with a uterus? It definitely says a lot about where my mindset was back then*

3. **Joy expressed in personal experiences with CBT (Spanish):**

- *I used to have frequent panic attacks and took antidepressants for a while, but what really helped me the most was cognitive-behavioral therapy (CBT)*

4. **Joy expressed in personal experiences with ACT (Spanish)**

- *"I'm really proud of a friend who is getting through a difficult period and, without even realising it, has been using Acceptance and Commitment Therapy techniques. I wasn't involved at all, but it's amazing to see how psychology can make such a difference."*

5. **Joy expressed in clinical indications of ACT (Spanish)**

- *" 🧐 Have you heard of Acceptance and Commitment Therapy (ACT)? ACT is an effective non-pharmacological approach for treating depression, helping people build a new way of relating to difficult or unwanted thoughts."*

6. **Joy expressed to psychoanalytic/psychodynamic therapy English:**

- *To break free from those "unconscious patterns," I found psychoanalysis incredibly valuable. I enjoy it because it feels scientific in its exploration of emotions and avoids being dogmatic.*

7. **Anger related to psychoanalytic/psychodynamic therapy (Spanish)**

- *"Everyone thinks they know what psychoanalysis is — except psychoanalysts themselves… because if we truly knew, contingency wouldn't exist."*

8. **Joy expressed in outreach and promotion of Narrative Therapy (Spanish)**

- *"Great response! Only one week left before the Narrative Therapy course begins! More information at…"*
